# Night-to-night sleep variability and all-cause and cardiovascular mortality: a prospective cohort study with a complementary *in vitro* circadian-disruption experiment

**DOI:** 10.3389/fmed.2026.1917205

**Published:** 2026-09-29

**Authors:** Jing Bai, Peng Chen, Chao Cui, Lilan Yang

**Affiliations:** 1 Department of Cardiology, Taiyuan Central Hospital, Peking University First Hospital Taiyuan Hospital, Ninth Clinical Medical College of Shanxi Medical University, Taiyuan Cardiovascular Disease Research Institute, Taiyuan, China; 2 Department of Imaging, Taiyuan Central Hospital, Peking University First Hospital Taiyuan Hospital, Ninth Clinical Medical College of Shanxi Medical University, Taiyuan Cardiovascular Disease Research Institute, Taiyuan, China

**Keywords:** accelerometry, all-cause mortality, circadian disruption, sleep regularity, sleep variability

## Abstract

**Background:**

Irregular sleep–wake patterns may disrupt circadian rhythms, but the dose–response relationship between night-to-night sleep variability and mortality and the association of sleep onset variability with death remain unclear.

**Methods:**

Using 4–7 valid days of wrist accelerometer data, including ≥1 weekend day, from 7,604 adults aged ≥20 years in the National Health and Nutrition Examination Survey 2011–2014, linked to mortality through December 2019, we examined the within-person SDs of sleep midpoint, sleep onset, and total sleep time across nights and the sleep regularity index (SRI) in relation to all-cause and cardiovascular disease (CVD) mortality. Survey-weighted Cox models were fitted across 20 imputed datasets and combined using Rubin's rules. Restricted cubic splines examined dose–response relationships. Complementary categorical, subgroup, and sensitivity analyses were conducted. Human umbilical vein endothelial cell (HUVEC) experiments repeated in three independent runs compared cellular responses under a regular dexamethasone schedule and four prespecified irregular schedule variants.

**Results:**

Over a median follow-up of 81 months, 714 all-cause and 229 CVD deaths occurred. Each 1-hour increase in the across-night SD of sleep midpoint, sleep onset, and total sleep time was associated with all-cause mortality HRs of 1.22 (95% CI, 1.12–1.33), 1.19 (1.11–1.29), and 1.17 (1.05–1.31), respectively. The corresponding CVD mortality HRs were 1.21 (1.09–1.35), 1.22 (1.05–1.42), and 1.16 (0.94–1.42), respectively. Each 10-point lower SRI was associated with all-cause mortality (HR, 1.29; 95% CI, 1.21–1.38) and CVD mortality (HR, 1.31; 95% CI, 1.18–1.45). Across three independent HUVEC runs, the mean response across the four prespecified irregular schedule variants showed lower core clock-gene expression, lower Griess assay absorbance, and higher inflammatory and adhesion-marker expression than the regular schedule.

**Conclusions:**

Greater night-to-night sleep irregularity was associated with higher hazards of all-cause and CVD mortality, although these associations were modest compared with those of older age, hypertension, and diabetes in the same models. The complementary cellular experiment showed differences between the regular dexamethasone schedule and the prespecified irregular schedule set.

## 1 Introduction

Sleep is a fundamental determinant of health (1). Both short and long sleep duration have been consistently associated with elevated risks of cardiovascular disease (CVD) (2), type 2 diabetes (3), and all-cause mortality in prospective studies (4, 5), and the American Heart Association has incorporated sleep duration as one of the eight core components of cardiovascular health in its Life's Essential 8 framework (6). Yet sleep is a multidimensional behavior, and how regularly a person sleeps from night to night may matter for long-term health independently of how long they sleep (7–9).

The biological basis for this possibility lies in circadian physiology. Blood pressure, heart rate, autonomic tone, glucose metabolism, and inflammatory responses all follow endogenous approximately 24-hour rhythms that are synchronized to the timing of the sleep–wake cycle (10–12). When an individual's sleep schedule varies substantially from day to day, these rhythms become misaligned with one another and with the external environment—a form of circadian disruption that is not limited to shift workers but is common in the general population (9, 13). Studies have shown that such misalignment, even without net sleep loss, acutely raises blood pressure, increases systemic inflammatory markers, and impairs glucose tolerance, providing a plausible pathway through which chronic sleep irregularity could increase mortality risk over time (14–16).

Consistent with this hypothesis, prospective cohort studies have reported that greater sleep–wake regularity, measured by composite indices or by the night-to-night variability of sleep duration and timing, is associated with lower risks of cardiovascular events and all-cause mortality (17–20). However, the shape of the continuous dose–response relationship between sleep variability and mortality has not been fully characterized (18, 20, 21). Prior prospective studies have examined sleep onset variability in relation to mortality, but evidence remains limited and has largely been derived from selected cohorts using heterogeneous exposure definitions and analytic approaches (17, 20). Whether these associations generalize to a nationally representative US population remains uncertain.

In this study, we used wrist accelerometer data from up to 7 consecutive days, requiring at least 4 valid days including 1 weekend day, in 7,604 US adults in National Health and Nutrition Examination Survey (NHANES) 2011–2014, linked to the National Death Index through December 31, 2019, to examine the associations of sleep midpoint variability, sleep onset variability, total sleep time variability, and SRI with all-cause and CVD mortality. In a complementary HUVEC experiment, we compared cellular responses under a regular dexamethasone schedule and four prespecified irregular schedule variants.

## 2 Methods

### 2.1 Study design and population

This prospective cohort study used data from the 2011–2012 and 2013–2014 cycles of the NHANES, a nationally representative, cross-sectional survey of the US civilian noninstitutionalized population conducted by the National Center for Health Statistics. All NHANES protocols received approval from the National Center for Health Statistics (NCHS) Ethics Review Board (ERB) (Protocol #2011-17). All methods were carried out in accordance with the Declaration of Helsinki. These two cycles were selected because they uniquely incorporated a 24-hour physical activity monitoring component in which participants wore wrist-mounted ActiGraph GT3X + accelerometers continuously for 7 consecutive days, enabling objective, minute-level classification of sleep and wake states.

From an initial pool of 19,931 participants across both cycles, we restricted the sample to adults aged 20 years or older (*n* = 11,329), applied the accelerometer quality criteria described below, and excluded participants whose sleep regularity index (SRI) or any of the three sleep-variability measures was more than 3 SDs from the corresponding sample mean, yielding 7,648 participants with ≥4 valid sleep days. After linkage to the National Death Index (NDI) through December 31, 2019, the final analytic cohort included 7,604 participants. All analyses incorporated the recommended 4-year combined examination sample weights and accounted for the complex multistage probability sampling design, including stratification and clustering at the primary sampling unit level.

### 2.2 Accelerometer-derived sleep metrics

The NHANES accelerometer component provides minute-level predictions of sleep–wake state, generated by a validated algorithm that classifies each epoch as wake or sleep (22, 23). Specifically, each 1-minute epoch was assigned the NHANES-derived sleep/wake label from the wrist-acceleration signal; contiguous sleep-labeled epochs were then processed as described below. A central challenge in using these data for sleep regularity research is the accurate delineation of nocturnal sleep periods when sleep episodes span midnight. To address this, we constructed a noon-anchored time axis in which each analytic sleep day ran from 12:00 PM to 11:59 AM the following day, ensuring that a single nocturnal sleep episode was not fragmented across two calendar dates (24).

Quality control was applied at both the day and person levels. A valid day required at least 960 min (16 h) of technically valid accelerometer data within a noon-to-noon window. Each participant was required to contribute at least 4 valid days, including at least 1 weekend day, to represent both weekday and weekend sleep patterns (25).

Within each valid day, the main sleep episode was identified through a stepwise procedure. Contiguous blocks of sleep-classified epochs were first detected. Brief awakenings shorter than 60 min that occurred between two sleep blocks were then bridged as intra-sleep arousals, preventing artificial fragmentation of the sleep period. The longest resulting sleep block within each noon-based day was designated as the main sleep episode. Episodes shorter than 2 h or longer than 18 h were excluded as biologically implausible.

Three night-to-night variability metrics were calculated for each participant across their valid nights. Sleep onset variability was defined as the within-person standard deviation (SD) of daily sleep onset clock times; sleep midpoint variability as the SD of daily sleep midpoint clock times; and total sleep time variability as the SD of daily sleep durations, each expressed in hours. For sleep onset and midpoint variability, circular statistics were used to handle the wrap-around nature of clock time at midnight. Each time was mapped onto a circular scale and the circular SD was computed from the mean resultant vector length (26). This approach avoids the spurious inflation of variability that would occur with conventional linear SD when sleep times cluster near midnight.

We also calculated the SRI for each participant (27). The SRI quantifies the epoch-by-epoch probability that an individual's sleep–wake state at any given minute is concordant with the state at the corresponding minute 24 h later, rescaled to range from −100 to 100. Higher SRI values reflect greater day-to-day consistency in sleep–wake patterns (27). Only epoch pairs with valid sleep/wake classifications at both time points contributed to the calculation; missing and non-wear epochs were excluded from both the numerator and denominator rather than classified as wake. For the actograms, clock time was reconstructed from the participant-specific first-data timestamp and the elapsed 80-Hz sample count. To illustrate the range of observed sleep–wake regularity, we selected four participants with 7 recorded days and less than 5% non-wear time. Eligible participants were ranked by inverse SRI, and those nearest the 10th, 35th, 65th, and 90th percentiles were selected. Because each evening-anchored window spanned two calendar days, six complete 24-hour windows (20:00 to 19:59 the following day) were displayed from each 7-day record.

### 2.3 Mortality ascertainment

Vital status was ascertained through probabilistic linkage with the NDI through December 31, 2019. Participants whose underlying cause of death was classified as accidents were excluded from the analytic cohort. The two primary outcomes were all-cause mortality and CVD mortality, with the latter defined by the underlying cause of death using International Classification of Diseases, 10th Revision codes for cardiovascular diseases (I00–I09, I11, I13, I20–I51, I60–I69). For cause-specific CVD models, non-CVD deaths were retained in the risk set and censored at the recorded time of death. Follow-up time was calculated in person-months from the mobile examination center visit to the date of death or the end of follow-up, whichever occurred first.

### 2.4 Covariate assessment

We adjusted for a comprehensive set of covariates selected on the basis of prior literature linking them to both sleep patterns and mortality (17–20). Demographic and socioeconomic variables included age (≤50 or >50 years), sex, race/ethnicity (non-Hispanic White, non-Hispanic Black, or other), educational level (less than 9th grade, 9th–12th grade or high school graduate/GED, or some college or above), marital status (married or living with partner vs divorced, separated, widowed, or never married), and the poverty-income ratio (PIR; <1.30, 1.30–3.50, or >3.50). Behavioral covariates included smoking status (current, former, or never) and alcohol consumption (current, former, or never). Body mass index (BMI) was calculated from measured height and weight and categorized as less than 25, 25 to 29.9, or 30 or greater. Hypertension was defined as self-reported physician diagnosis, measured systolic blood pressure of 140 mm Hg or higher, measured diastolic blood pressure of 90 mm Hg or higher, or current use of antihypertensive medication. Diabetes was defined as self-reported physician diagnosis, glycated hemoglobin of 6.5% or higher, fasting plasma glucose of 7.0 mmol/L or higher, 2-hour oral glucose tolerance test glucose of 11.1 mmol/L or higher, or current use of antidiabetic medication. Baseline cancer was defined by self-reported physician diagnosis.

### 2.5 *In vitro* dexamethasone-schedule model and endothelial assessment

Human umbilical vein endothelial cells (HUVECs; ScienCell Research Laboratories, Carlsbad, CA, USA; catalog no. 8000) were cultured in endothelial cell medium basal (ScienCell, 1001-b) supplemented with 5% fetal bovine serum (ScienCell, 0025), 1% endothelial cell growth supplement (ScienCell, 1052), and 1% penicillin/streptomycin solution (ScienCell, 0503) at 37 °C in a humidified atmosphere of 5% CO₂. Cells at early passages (P3–P6) were used. The full protocol was repeated in three independently initiated experimental runs. Each run included four wells receiving the same regular schedule and one well for each of four prespecified irregular schedule variants. Dexamethasone can synchronize or phase-reset circadian gene expression in cultured peripheral cells, and irregular dexamethasone stimulation cycles have previously been used to model cellular circadian disruption (28, 29). In every run, every well received seven 100 nM dexamethasone (Sigma-Aldrich, D4902) exposures of 2 h each between 0 and 168 h. After each exposure, cells were washed once with PBS and returned to dexamethasone-free medium. All regular-group wells were stimulated at 0, 24, 48, 72, 96, 120, and 144 h, whereas the four irregular-group wells received four distinct prespecified schedules with successive intervals of 16–32 h (Supplementary Table S1). All samples were collected at the common endpoint of 168 h. Cell viability was assessed using the Cell Counting Kit-8 assay (Beyotime, C0038), with blank-corrected absorbance measured at 450 nm. The nitrite-related Griess reaction signal was assessed using a nitric oxide assay kit (Beyotime, S0021), with blank-corrected absorbance measured at 540 nm. Total RNA was extracted using TRIzol (Invitrogen, 15596026), and relative mRNA expression of BMAL1, PER2, IL-6, TNF-α, ICAM-1, and VCAM-1 was quantified using the 2^⁻*ΔΔ*^Ct method with a qPCR master mix (Vazyme, Q711) and GAPDH as the internal reference. Within each run, the four identically scheduled regular wells were averaged to obtain the regular-condition summary, whereas the four prespecified irregular schedule variants were averaged to summarize the response across the irregular-schedule set.

### 2.6 Statistical analysis

Missing covariate data were below 10% (range, 0%–7.3%; Supplementary Table S2) and were handled using multiple imputation by chained equations (R package mice). Twenty completed datasets were generated using 20 iterations with predictive mean matching. Imputed variables were educational level, marital status, PIR, alcohol consumption, smoking status, BMI, and diabetes. Survey-weighted Cox models were fitted separately in all 20 completed datasets, and regression coefficients and within-imputation variances were combined using Rubin's rules. Baseline descriptive summaries used the first completed dataset. Baseline characteristics were summarized as unweighted counts with survey-weighted percentages for categorical variables and survey-weighted means with SDs for continuous variables. Differences between mortality groups were compared using survey-weighted chi-square tests and *t* tests.

Survey-weighted Cox proportional hazards models were used to estimate hazard ratios (HRs) and 95% confidence intervals (CIs). Each sleep-variability exposure represented the within-person SD of the corresponding sleep measure across nights and was modeled per 1-hour increase. Under the proportional hazards assumption, an HR represents the relative instantaneous hazard of death associated with a specified exposure difference among participants still at risk; it does not represent a direct difference in the probability or absolute risk of death. The principal clinically interpretable SRI estimate was modeled per 10-point lower value; per-point estimates were retained in Supplementary Table S5. Three progressively adjusted models were constructed: Model 0, unadjusted; Model 1, adjusted for age, sex, and race/ethnicity; and Model 2, additionally adjusted for educational level, marital status, PIR, alcohol consumption, smoking status, BMI, hypertension, and diabetes. All categorical covariates were entered as factors. The proportional hazards assumption was assessed using interactions between each sleep measure and follow-up period (<60 vs. ≥60 months). Complete coefficient estimates for all terms in Model 2, including the reference categories for categorical covariates, are provided in Supplementary Table S5.

To explore potential nonlinear dose–response relationships, restricted cubic splines with 3 degrees of freedom were fitted within Model 2. The two internal knots were placed at the 33.3rd and 66.7th percentiles of each exposure, with boundary knots at the observed minimum and maximum. This parsimonious specification allowed nonlinearity while limiting overfitting for the smaller CVD endpoint. The observed minimum served as the reference (HR = 1.0), and HRs were plotted across the 1st to 99th percentile range. Overall and nonlinearity Wald *P* values were reported. All restricted cubic spline models were fitted separately in the 20 completed datasets, with coefficients and covariance matrices combined using Rubin's rules.

To facilitate interpretation, complementary categorical analyses compared sleep onset variability >90 min with ≤90 min, informed by a previous prospective study in which sleep-onset timing variability >90 min was associated with higher cardiovascular risk (19). SRI was categorized as <70, 70–79, and ≥80 based on published population distributions (30), and was additionally modeled as <70 versus ≥70. Because the ≥80 category was uncommon in this cohort, categorical estimates were interpreted cautiously and the continuous estimates remained primary. Survey-weighted distributions of the continuous sleep metrics and corresponding categories were tabulated.

Stratified analyses were conducted within Model 2 to evaluate potential effect modification by age (≤50 vs. >50 years), sex, race/ethnicity, educational level, marital status, PIR, alcohol consumption, smoking status, BMI, hypertension, and diabetes. *P* values for interaction were computed from multiplicative interaction terms and summarized both before and after Benjamini–Hochberg correction. For the strongest nominal interaction signals, secondary analyses described marital-status and smoking-status patterns using stratum-specific HRs per 1-SD higher exposure and joint marital-status-by-smoking-status strata. All imputation-dependent coefficients and variances were combined using Rubin's rules. These subgroup analyses were considered exploratory.

Seven sensitivity analyses were conducted to assess Model 2: (1) additional adjustment for accelerometer valid recording days; (2) additional adjustment for participant-level mean sleep duration across valid sleep periods; (3) additional adjustment for NHANES survey cycle; (4) exclusion of participants with less than 1 year of follow-up; (5) exclusion of participants with baseline cancer; (6) complete-case analysis; and (7) restriction to participants with all 7 valid recording days. Analyses using imputed covariates were pooled across the 20 datasets; the complete-case analysis was fitted once. To compare effect sizes on a common scale, we also estimated HRs per 1-SD higher sleep variability, per 1-SD lower SRI, and per 1-SD higher mean sleep duration. Approximate E-values, treating HRs as risk-ratio approximations, were calculated from the fully adjusted estimates and confidence limits nearest the null to assess sensitivity to unmeasured confounding (31).

For the *in vitro* experiments, the independent experimental run was the unit of inference (*n* = 3). Within each run, the four identically scheduled regular wells were averaged to obtain the regular-condition summary, whereas the four prespecified irregular schedule variants were averaged to obtain the irregular-schedule-set summary. The three paired run-level summaries were used for inference; the irregular schedules were not treated as replicate wells receiving an identical exposure. For qPCR, target and GAPDH Cq values were averaged within condition and run, and two-sided one-sample t tests compared run-level *ΔΔ*Ct values with 0. For plotting, expression was normalized to a gene-specific common calibrator defined as the mean *Δ*Ct of the regular group across the three runs, allowing between-run variation to remain visible. Statistical inference was based on the run-level *ΔΔ*Ct values and was unaffected by this plotting transformation. For CCK-8 and Griess assays, blank-corrected condition means were calculated in each run and two-sided one-sample t tests compared the run-level paired differences with 0. Results are presented as mean ± SD across the three independent runs.

All analyses were performed using R (4.3.2), with a 2-sided *P* < 0.05 considered statistically significant.

## 3 Results

### 3.1 Study population and baseline characteristics

Among the 7,604 participants in the final analytic cohort, 714 all-cause deaths and 229 CVD deaths occurred over a median follow-up of 81 months (interquartile range, 69–94 months; range, 1–113 months). A total of 247 (survey-weighted 3.1%), 837 (10.9%), 3,170 (41.3%), and 3,350 (44.6%) participants contributed 4, 5, 6, and 7 valid recording days, respectively (Supplementary Table S3). Table 1 presents baseline characteristics by all-cause mortality status, and Supplementary Table S4 presents the corresponding data by CVD mortality.

**Table 1:** Baseline characteristics of study participants stratified by all-cause mortality status.

Characteristic	Category	Overall	Alive (*n* = 6,890)	Died (*n* = 714)	*P value*
Age	<=50 years	3,788 (53.1%)	3,723 (56.6%)	65 (10.6%)	<0.001
	>50 years	3,816 (46.9%)	3,167 (43.4%)	649 (89.4%)	
Sex	Male	3,614 (46.9%)	3,245 (46.8%)	369 (48.2%)	0.494
	Female	3,990 (53.1%)	3,645 (53.2%)	345 (51.8%)	
Race and ethnicity	Non-Hispanic White	3,111 (67.4%)	2,701 (66.5%)	410 (78.3%)	<0.001
	Non-Hispanic Black	1,769 (11.0%)	1,606 (11.0%)	163 (10.6%)	
	Other	2,724 (21.6%)	2,583 (22.5%)	141 (11.1%)	
Educational level	Less than 9th grade	667 (5.1%)	561 (4.7%)	106 (10.2%)	<0.001
	9th−12th grade or high school graduate/GED	2,670 (31.4%)	2,371 (30.8%)	299 (38.9%)	
	Some college or above	4,267 (63.5%)	3,958 (64.5%)	309 (50.9%)	
Marital status	Married/partnered	4,457 (63.5%)	4,122 (64.6%)	335 (51.2%)	<0.001
	Other	3,147 (36.5%)	2,768 (35.4%)	379 (48.8%)	
Poverty-income ratio	<1.30	2,618 (23.5%)	2,316 (22.8%)	302 (31.5%)	<0.001
	1.30–3.50	2,651 (35.2%)	2,372 (34.6%)	279 (42.4%)	
	>3.50	2,335 (41.3%)	2,202 (42.6%)	133 (26.1%)	
Alcohol consumption	Current	5,439 (77.5%)	4,954 (78.1%)	485 (71.0%)	0.019
	Former	969 (10.1%)	856 (9.8%)	113 (14.0%)	
	Never	1,196 (12.3%)	1,080 (12.1%)	116 (15.1%)	
Smoking status	Current	1,414 (18.2%)	1,282 (18.1%)	132 (19.3%)	<0.001
	Former	1,837 (25.2%)	1,559 (24.1%)	278 (38.9%)	
	Never	4,353 (56.5%)	4,049 (57.8%)	304 (41.8%)	
Body mass index	<25 kg/m2	2,246 (28.4%)	2,021 (28.3%)	225 (29.6%)	0.701
	25–29.9 kg/m2	2,453 (33.4%)	2,230 (33.5%)	223 (31.9%)	
	>=30 kg/m2	2,905 (38.2%)	2,639 (38.2%)	266 (38.5%)	
Hypertension	No	4,240 (59.9%)	4,075 (62.8%)	165 (25.0%)	<0.001
	Yes	3,364 (40.1%)	2,815 (37.2%)	549 (75.0%)	
Diabetes	No	6,141 (85.3%)	5,709 (86.9%)	432 (65.9%)	<0.001
	Yes	1,463 (14.7%)	1,181 (13.1%)	282 (34.1%)	
Sleep midpoint variability, h		1.39 (0.94)	1.37 (0.92)	1.65 (1.04)	<0.001
Total sleep time variability, h		1.63 (0.71)	1.61 (0.70)	1.80 (0.77)	<0.001
Sleep onset variability, h		1.60 (0.93)	1.58 (0.93)	1.85 (1.00)	<0.001
Sleep Regularity Index, points		61.96 (14.95)	62.66 (14.64)	53.43 (16.04)	<0.001

Data are presented as unweighted counts with survey-weighted percentages for categorical variables and survey-weighted means (standard deviations) for continuous variables. Baseline summaries used the first completed dataset. Sleep midpoint, sleep onset, and total sleep time variability are participant-level within-person SDs of the corresponding measures across valid nights. *P* values were calculated using survey-weighted chi-square tests or *t* tests. The analytic cohort required ≥4 valid recording days and included 7,604 participants. BMI, body mass index; PIR, poverty-income ratio; SRI, sleep regularity index.

Compared with survivors, participants who died during follow-up were older (89.4% vs. 43.4% aged >50 years), more likely to be non-Hispanic White (78.3% vs. 66.5%), less likely to have some college education or above (50.9% vs. 64.5%) or to be married or partnered (51.2% vs. 64.6%), and had lower PIR and higher prevalences of hypertension and diabetes (all *P* < 0.001). Alcohol-consumption and smoking-status distributions also differed between the groups (*P* = 0.019 and *P* < 0.001, respectively). All three sleep variability metrics were elevated among decedents: mean sleep midpoint variability was 1.65 h (SD, 1.04) versus 1.37 h (SD, 0.92); total sleep time variability was 1.80 h (SD, 0.77) versus 1.61 h (SD, 0.70); and sleep onset variability was 1.85 h (SD, 1.00) versus 1.58 h (SD, 0.93; all *P* < 0.001). Mean SRI was also lower among decedents (53.43 vs. 62.66; *P* < 0.001).

### 3.2 Association of sleep variability with mortality

In survey-weighted Cox regression analyses pooled across the 20 imputed datasets, the sleep-variability metrics showed graded associations with mortality (Figure 1). In Model 2, each 1-hour increase in the across-night SD of sleep midpoint was associated with higher hazards of all-cause mortality (HR, 1.22; 95% CI, 1.12–1.33; *P* < 0.001) and CVD mortality (HR, 1.21; 95% CI, 1.09–1.35; *P* < 0.001). Each 1-hour increase in the across-night SD of sleep onset was associated with all-cause mortality (HR, 1.19; 95% CI, 1.11–1.29; *P* < 0.001) and CVD mortality (HR, 1.22; 95% CI, 1.05–1.42; *P* = 0.009). Each 1-hour increase in the across-night SD of total sleep time was associated with all-cause mortality (HR, 1.17; 95% CI, 1.05–1.31; *P* = 0.005). For CVD mortality, its HR was 1.16 (95% CI, 0.94–1.42; *P* = 0.164); the confidence interval included both the null and positive associations, leaving uncertainty about the true magnitude. The smaller number of CVD deaths limited precision but does not establish the absence of an association. In the complete Model 2 estimates, older age, hypertension, and diabetes had larger point estimates than the sleep-variability measures. Complete estimates for the sleep exposure and all factor-coded covariates are provided in Supplementary Table S5.

**Figure 1:**
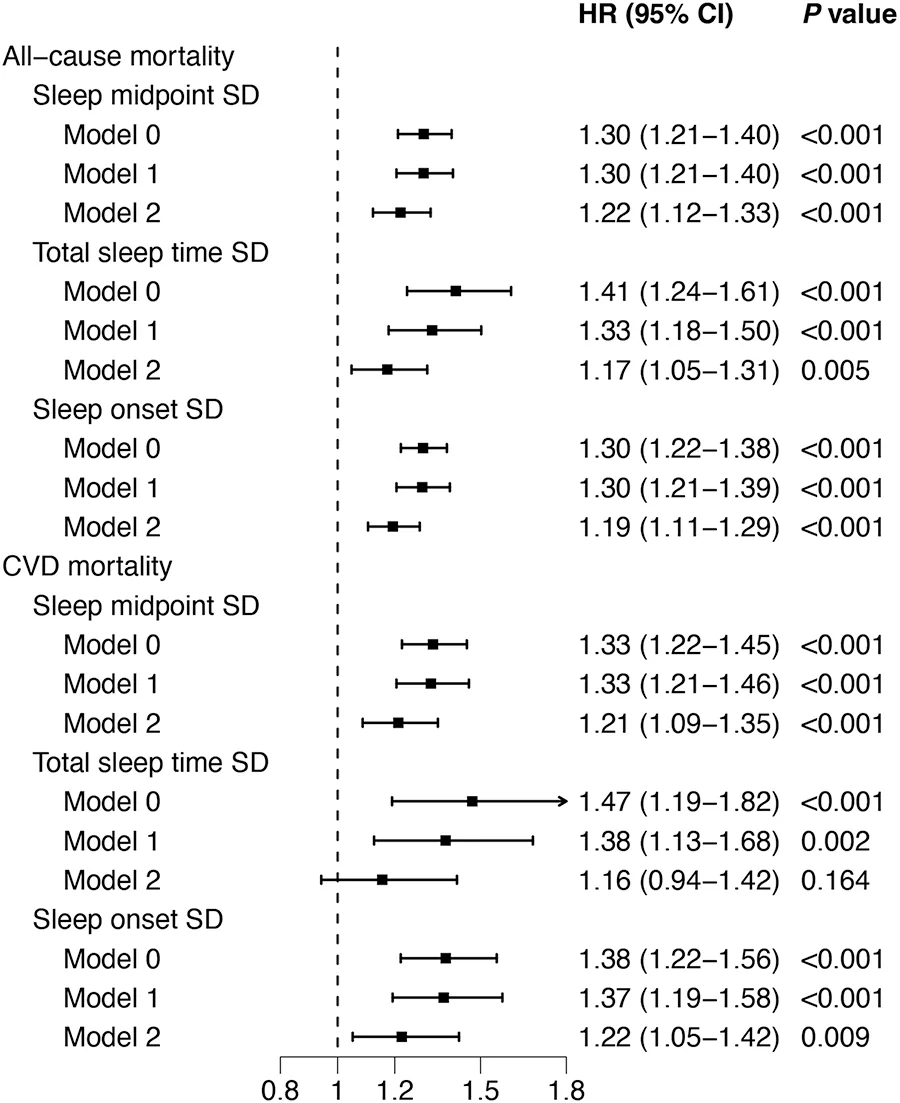
Associations of across-night sleep variability with all-cause and cardiovascular disease mortality. Forest plot showing hazard ratios (HRs) and 95% confidence intervals (CIs) for all-cause mortality (714 events) and cardiovascular disease (CVD) mortality (229 events) among 7,604 participants. Estimates are reported per 1-hour increase in the within-person standard deviation (SD) of each sleep measure across nights. Three sequential models are presented: Model 0, unadjusted; Model 1, adjusted for age, sex, and race/ethnicity; and Model 2, additionally adjusted for educational level, marital status, poverty-income ratio (PIR), alcohol consumption, smoking status, body mass index (BMI), hypertension, and diabetes. The dashed vertical line indicates HR = 1.0.

### 3.3 Dose–response relationships between sleep variability and mortality

Restricted cubic spline analyses showed overall associations of sleep midpoint, total sleep time, and sleep onset variability with all-cause mortality (Figures 2A–C; overall *P* < 0.001, *P* = 0.021, and *P* < 0.001, respectively). None showed evidence of nonlinearity (*P* > 0.05), supporting interpretation of the continuous estimates without a data-derived threshold.

**Figure 2:**
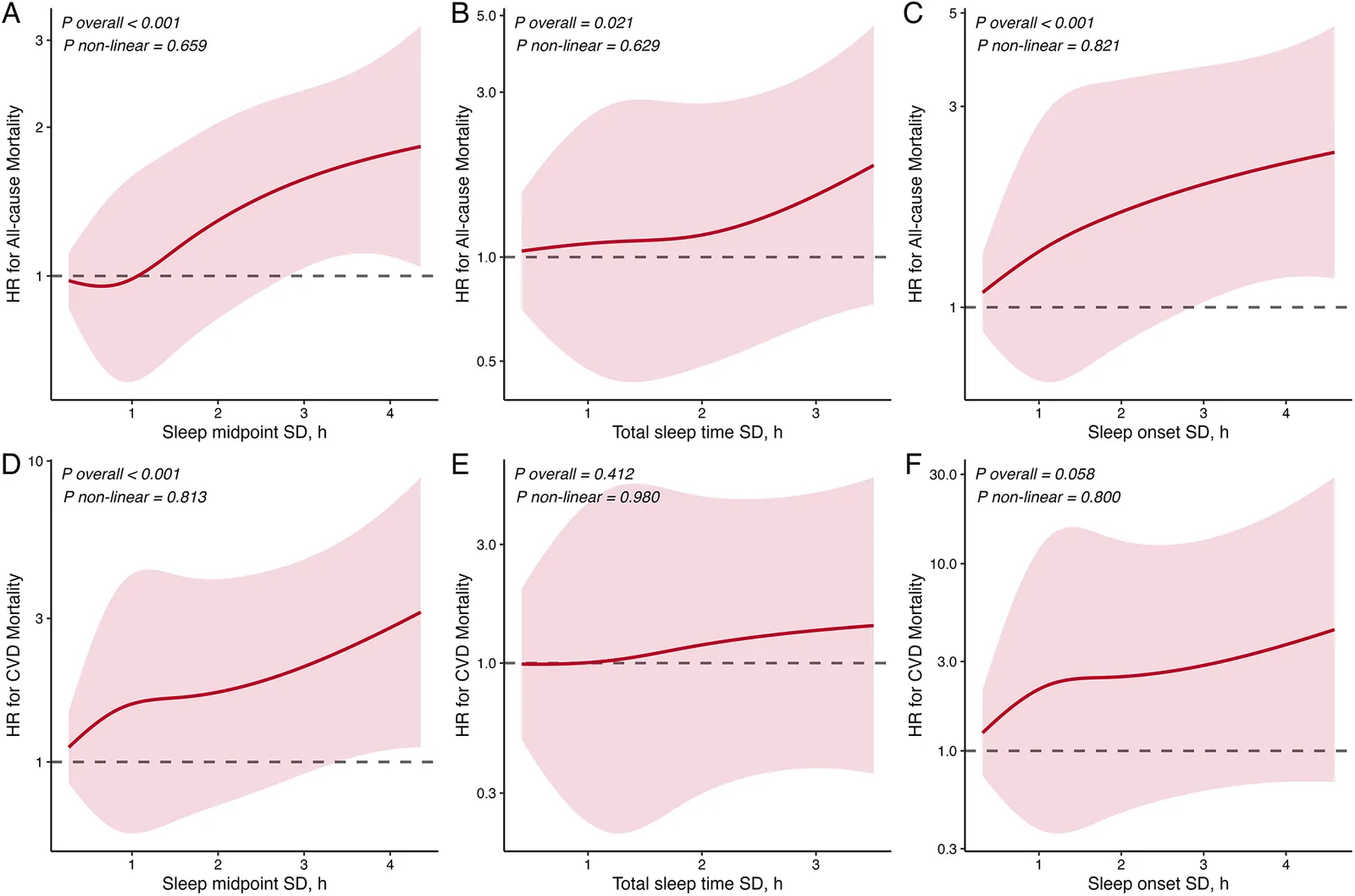
Dose–response relationships of across-night sleep variability with all-cause and cardiovascular disease mortality. Restricted cubic spline curves with 3 degrees of freedom based on model 2, depicting associations with all-cause mortality (714 events; A–C) and cardiovascular disease (CVD) mortality (229 events; D–F) among 7,604 participants. The exposures were the within-person standard deviations (SDs) of sleep midpoint, total sleep time, and sleep onset across nights, expressed in hours. **(A,D)** Sleep midpoint SD; **(B,E)** total sleep time SD; and **(C,F)** sleep onset SD. The observed minimum served as the reference, with a hazard ratio (HR) of 1.0, and curves are shown from the 1st to the 99th percentile. Solid lines represent HRs, shaded areas represent 95% confidence intervals (CIs), and the horizontal dashed line indicates HR = 1.0. Models were fitted separately in each of the 20 imputed datasets and combined using Rubin's rules.

For CVD mortality (Figures 2D–F), sleep midpoint variability showed an overall association (*P* < 0.001), whereas total sleep time and sleep onset variability did not (*P* > 0.05). None of the three curves showed evidence of nonlinearity (*P* > 0.05).

In the complementary categorical analysis, a within-person sleep onset SD >90 min, compared with ≤90 min, was associated with a higher hazard of all-cause mortality (HR, 1.29; 95% CI, 1.05–1.59; *P* = 0.015). For CVD mortality, the estimate was 1.23 (95% CI, 0.90–1.68; *P* = 0.198); the confidence interval included both the null and potentially meaningful positive associations, indicating substantial uncertainty about the magnitude of the association (Supplementary Table S6).

### 3.4 Sleep regularity index and mortality

We next examined SRI as a complementary measure of day-to-day sleep–wake regularity. SRI values were higher among survivors than among decedents (Figure 3A). Figure 3B shows four observed 7-day sleep–wake records selected near the 10th, 35th, 65th, and 90th percentiles of inverse SRI, with SRI decreasing from 78.0 at the 10th percentile to 40.8 at the 90th percentile. The dimension-specific SDs shown above each actogram illustrate that SRI and individual timing or duration metrics capture related but nonidentical features of sleep regularity. Survey-weighted proportions were 65.8% for SRI <70, 25.2% for SRI 70–79, and 8.9% for SRI ≥80; the corresponding unweighted counts were 5,353, 1,717, and 534 participants (Supplementary Table S6).

**Figure 3:**
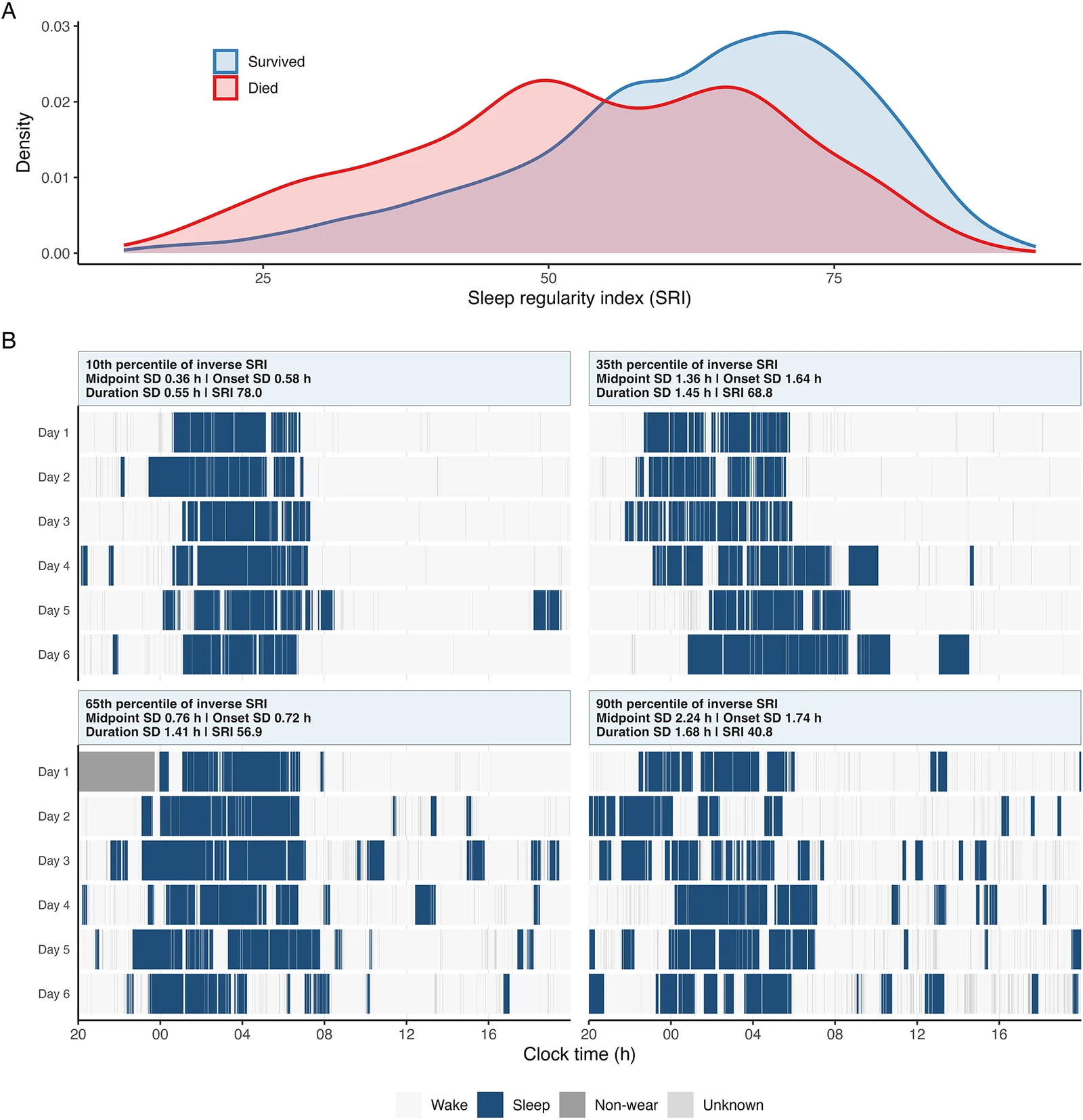
Distribution of sleep regularity and representative sleep–wake patterns. **(A)** survey-weighted density curves showing the distribution of the sleep regularity index (SRI) among participants who survived (blue; *n* = 6,890) and those who died from any cause (red; *n* = 714) during follow-up. **(B)** Minute-level sleep–wake actograms for four observed participants with 7 recorded days and less than 5% non-wear time, selected nearest the 10th, 35th, 65th, and 90th percentiles of inverse SRI among eligible participants. Because each evening-anchored window spans two calendar days, six complete 24-hour windows, running from 20:00 to 19:59 the following day, are displayed from each 7-day record. Clock time was reconstructed from the participant-specific first-data timestamp and elapsed sample count. Dark blue indicates sleep, near-white indicates wake, dark gray indicates non-wear, and light gray indicates unknown state. The within-person standard deviations (SDs) of sleep midpoint, sleep onset, and total sleep time across nights and the SRI are displayed for each participant. The four records are illustrative examples selected from the observed cohort.

In fully adjusted Cox models, each 10-point lower SRI was associated with higher hazards of all-cause mortality (HR, 1.29; 95% CI, 1.21–1.38; *P* < 0.001) and CVD mortality (HR, 1.31; 95% CI, 1.18–1.45; *P* < 0.001) (Figure 4A and Supplementary Table S6). Compared with SRI ≥80, the HRs for all-cause mortality were 1.73 (95% CI, 0.84–3.55; *P* = 0.138) for SRI 70–79 and 3.66 (95% CI, 1.91–7.02; *P* < 0.001) for SRI <70. Corresponding CVD mortality HRs were 2.21 (95% CI, 0.67–7.30; *P* = 0.194) and 5.28 (95% CI, 1.77–15.68; *P* = 0.003). The small number of events in the ≥80 reference group (17 all-cause and 4 CVD deaths) produced wide confidence intervals and warrants cautious interpretation.

**Figure 4:**
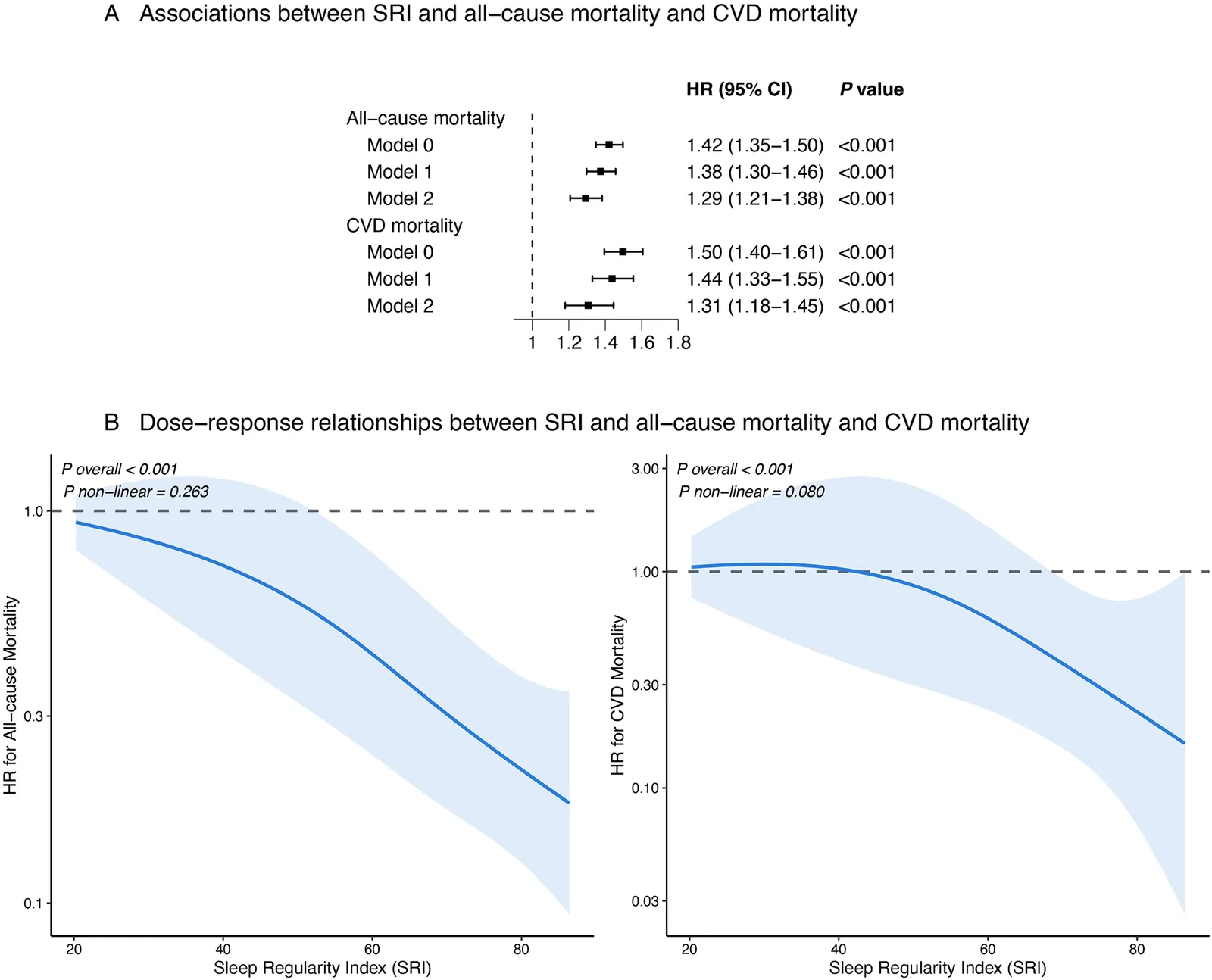
Sleep regularity index and mortality hazards. **(A)** forest plot showing Rubin-pooled hazard ratios (HRs) and 95% confidence intervals (CIs) for all-cause mortality (714 events) and cardiovascular disease (CVD) mortality (229 events) per 10-point lower sleep regularity index (SRI) among 7,604 participants. Model 0 was unadjusted; Model 1 was adjusted for age, sex, and race/ethnicity; and Model 2 was additionally adjusted for educational level, marital status, poverty-income ratio, alcohol consumption, smoking status, body mass index, hypertension, and diabetes. **(B)** Restricted cubic spline curves for all-cause mortality (left) and CVD mortality (right), fitted using Model 2 with 3 degrees of freedom and pooled across 20 imputed datasets using Rubin's rules. The observed minimum served as the reference (HR = 1.0); solid lines indicate HRs, shaded areas indicate 95% CIs, and the horizontal dashed line indicates HR = 1.0.

Restricted cubic spline analyses supported inverse dose–response relationships between SRI and both all-cause mortality (overall *P* < 0.001; nonlinearity *P* = 0.263) and CVD mortality (overall *P* < 0.001; nonlinearity *P* = 0.080) (Figure 4B).

### 3.5 Subgroup analyses

We then examined whether the observed associations differed across 11 prespecified demographic and clinical subgroups (Supplementary Table S7). Several raw interaction *P* values were below 0.05. Two of the 66 interaction tests remained statistically significant after Benjamini–Hochberg adjustment: the interactions of sleep midpoint and sleep onset variability with marital status for CVD mortality (adjusted *P* = 0.015 and *P* = 0.049, respectively). All other adjusted *P* values were ≥0.05, and the subgroup patterns were otherwise treated as exploratory.

For all-cause mortality, the standardized HR for sleep onset variability was 1.28 (95% CI, 1.13–1.45) among married or partnered participants and 1.09 (95% CI, 1.00–1.18) among other participants (raw *P* for interaction = 0.031; adjusted *P* = 0.408). The corresponding midpoint estimates were 1.28 (95% CI, 1.14–1.45) and 1.12 (95% CI, 1.03–1.23), but the raw interaction *P* value was 0.053 (Supplementary Table S8).

For CVD mortality, the interactions of sleep midpoint and sleep onset variability with marital status remained statistically significant after correction (raw *P* < 0.001 and *P* = 0.001; adjusted *P* = 0.015 and *P* = 0.049, respectively). Standardized HRs for sleep midpoint variability were 1.44 (95% CI, 1.23–1.69) among married or partnered participants and 1.01 (95% CI, 0.89–1.15) among other participants. The smoking-status interactions did not remain significant after correction (Supplementary Table S8). Joint marital-status-by-smoking-status estimates are provided in Supplementary Table S10 and are considered hypothesis-generating.

### 3.6 Sensitivity analyses

To evaluate the robustness of the primary findings, we conducted seven sensitivity analyses. Primary all-cause mortality estimates were similar after additional adjustment for valid recording days, participant mean sleep duration, or NHANES cycle; after exclusion of early deaths or baseline cancer; and in complete-case analyses (Supplementary Table S9). In the 7-day subset (*n* = 3,350; 389 all-cause and 127 CVD deaths), all-cause associations remained directionally consistent, whereas CVD estimates were attenuated and had wider confidence intervals. When expressed per 1-SD difference, lower SRI and greater variability in sleep midpoint and sleep onset were associated with higher mortality hazards, whereas mean sleep duration was not linearly associated with either outcome (Supplementary Table S11). Approximate E-values for statistically significant primary estimates ranged from 1.62 to 1.94 for the point estimates and from 1.27 to 1.71 for the confidence limits nearest the null (Supplementary Table S12). Interactions between each sleep measure and follow-up period (<60 vs. ≥60 months) were not statistically significant for either outcome (all *P* ≥ 0.05).

### 3.7 Cellular responses under regular and irregular dexamethasone schedules

To complement the population analyses, we compared clock-gene expression, CCK-8 and Griess assay absorbance, and inflammatory and adhesion-marker expression between the regular schedule and the prespecified irregular schedule set.

Across the three independent runs, the mean irregular-to-regular expression ratio was 0.44 ± 0.03 for BMAL1 (*P* = 0.003) and 0.47 ± 0.03 for PER2 (*P* = 0.002) (Figure 5).

**Figure 5:**
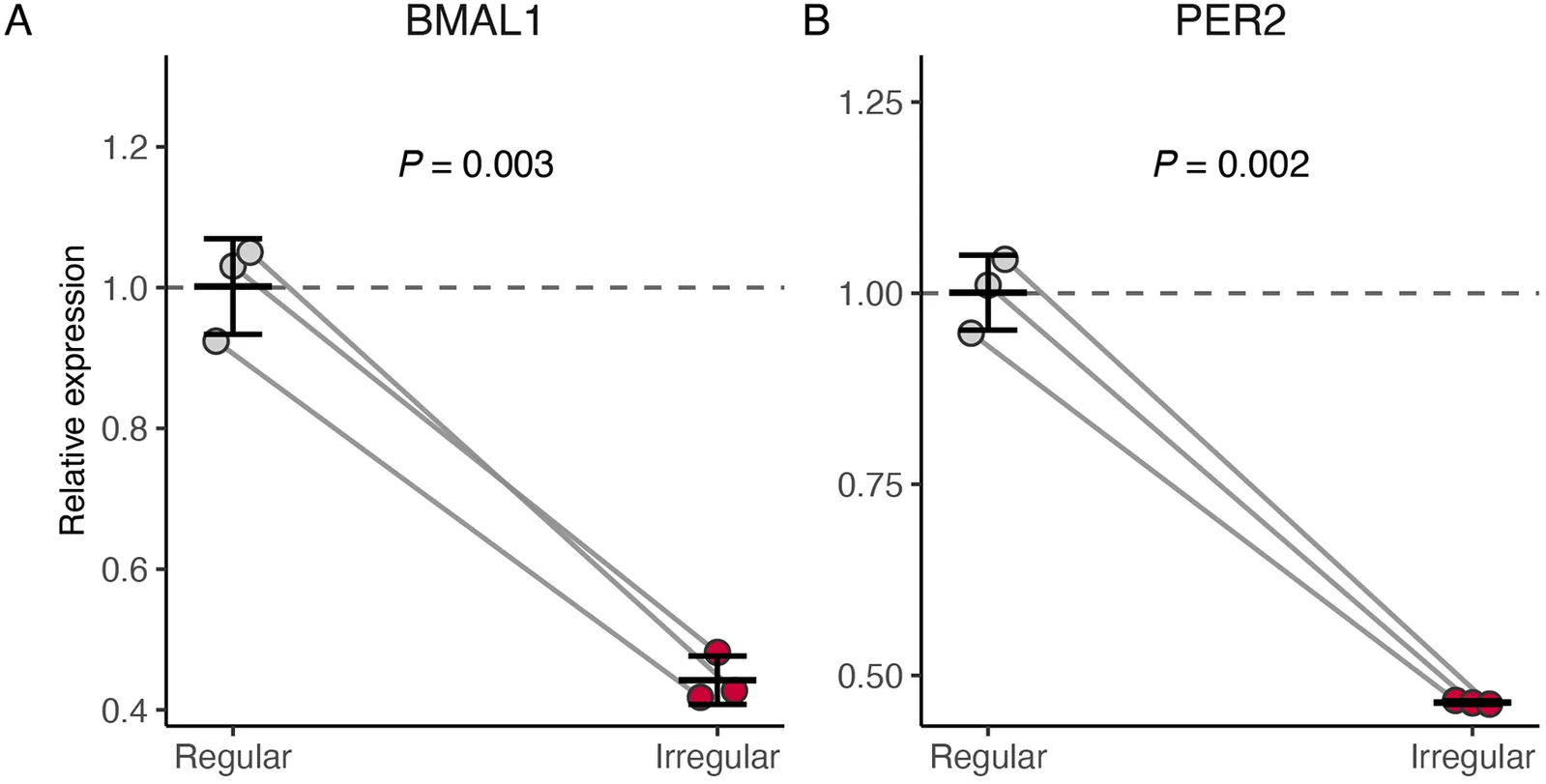
Core clock-gene expression under regular and irregular dexamethasone schedules. Relative messenger RNA (mRNA) expression of **(A)** BMAL1 and **(B)** PER2 in human umbilical vein endothelial cells (HUVECs) subjected to the regular 24-hour dexamethasone schedule or the four prespecified irregular schedule variants with successive intervals of 16–32 h. Each point represents the run-level condition summary from one independent experimental run (*n* = 3). Within each run, the regular summary was the mean of four identically scheduled wells, whereas the irregular summary was the mean response across four prespecified schedule variants. Points from the same run are connected by gray lines; black horizontal bars and error bars indicate the mean ± standard deviation (SD) across runs. Expression values were normalized to one gene-specific common calibrator, defined as the mean *Δ*Ct of the regular condition across all three runs, thereby preserving run-to-run variation in the regular condition. Ct denotes the cycle threshold. The dashed line indicates relative expression = 1. *P* values were calculated using two-sided one-sample *t* tests of the run-level *ΔΔ*Ct values against 0. The experiment was limited to three independent runs with common-endpoint sampling; schedule-related and phase-related effects therefore could not be separated.

We next assessed blank-corrected CCK-8 and Griess assay absorbance. Across runs, blank-corrected CCK-8 absorbance at 450 nm was 0.60 ± 0.03 under the regular schedule and 0.53 ± 0.03 for the irregular-schedule set (*P* = 0.003), corresponding to 87.88 ± 1.68% of the regular-schedule value. Blank-corrected Griess absorbance at 540 nm was 0.43 ± 0.03 under the regular schedule and 0.20 ± 0.03 for the irregular-schedule set (*P* < 0.001) (Figure 6).

**Figure 6:**
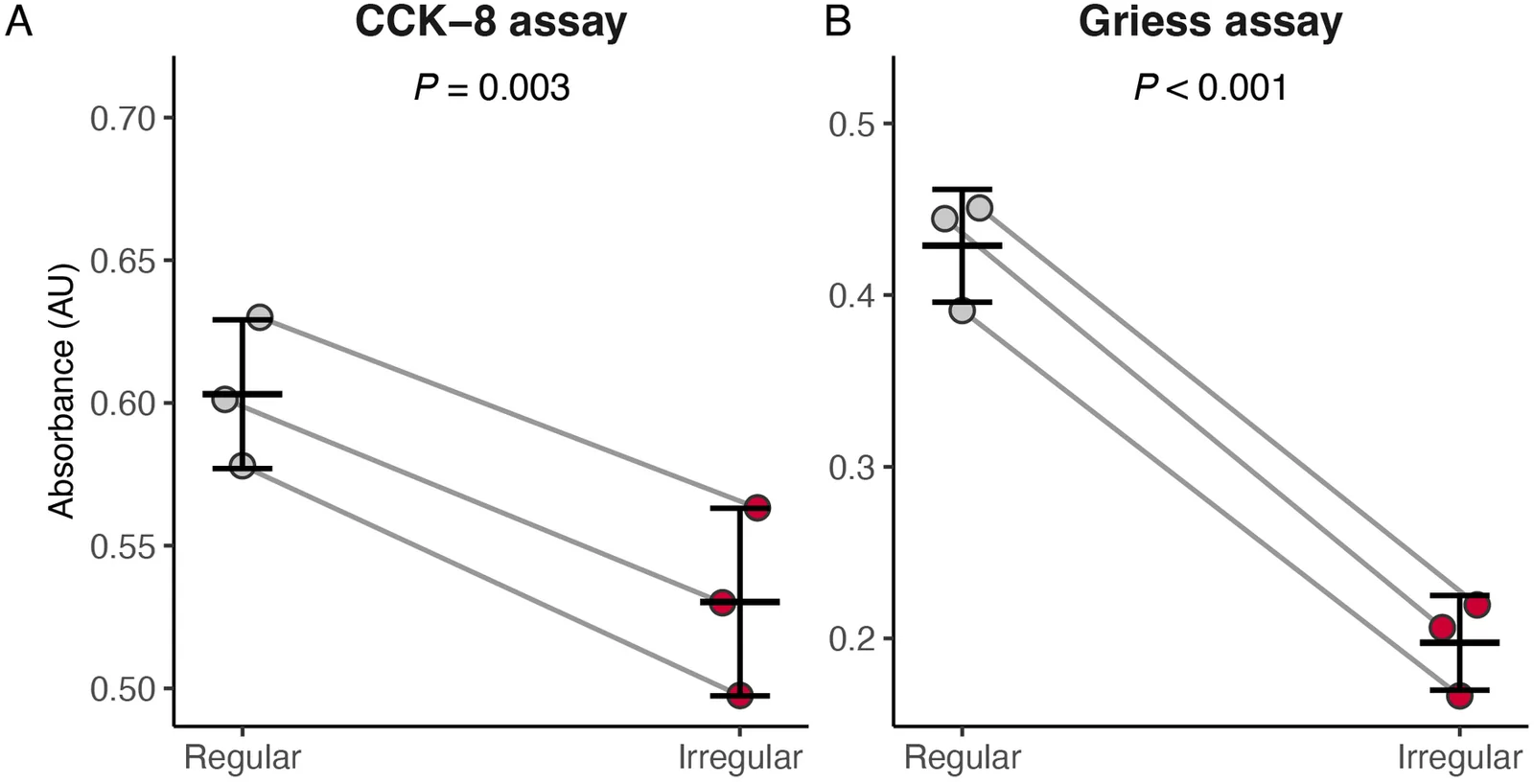
CCK-8 and Griess assay absorbance under regular and irregular dexamethasone schedules. **(A)** Cell Counting Kit-8 (CCK-8) absorbance and **(B)** the nitrite-related Griess reaction signal in human umbilical vein endothelial cells (HUVECs) subjected to the regular dexamethasone schedule or the four prespecified irregular schedule variants. Each point represents the run-level condition summary from one independent experimental run (*n* = 3). Within each run, the regular summary was the mean of four identically scheduled wells, whereas the irregular summary was the mean response across four prespecified schedule variants. Points from the same run are connected by gray lines; black horizontal bars and error bars indicate the mean ± standard deviation (SD) across runs. Values are blank-corrected absorbance at 450 nm for CCK-8 and 540 nm for the Griess assay. *P* values were calculated using two-sided one-sample *t* tests of the run-level paired differences against 0. The experiment was limited to three independent runs with common-endpoint sampling; schedule-related and phase-related effects therefore could not be separated. The Griess result was analyzed as blank-corrected absorbance without a nitrite standard curve or normalization to cell number, protein, or DNA and should therefore be interpreted as semiquantitative.

Finally, we evaluated inflammatory and endothelial adhesion markers. Across the three independent runs, the mean irregular-to-regular expression ratios were 2.71 ± 0.23 for IL-6 (*P* = 0.002), 1.92 ± 0.09 for TNF-α (*P* = 0.002), 2.80 ± 0.25 for ICAM-1 (*P* = 0.003), and 2.20 ± 0.13 for VCAM-1 (*P* = 0.002) (Figure 7).

**Figure 7:**
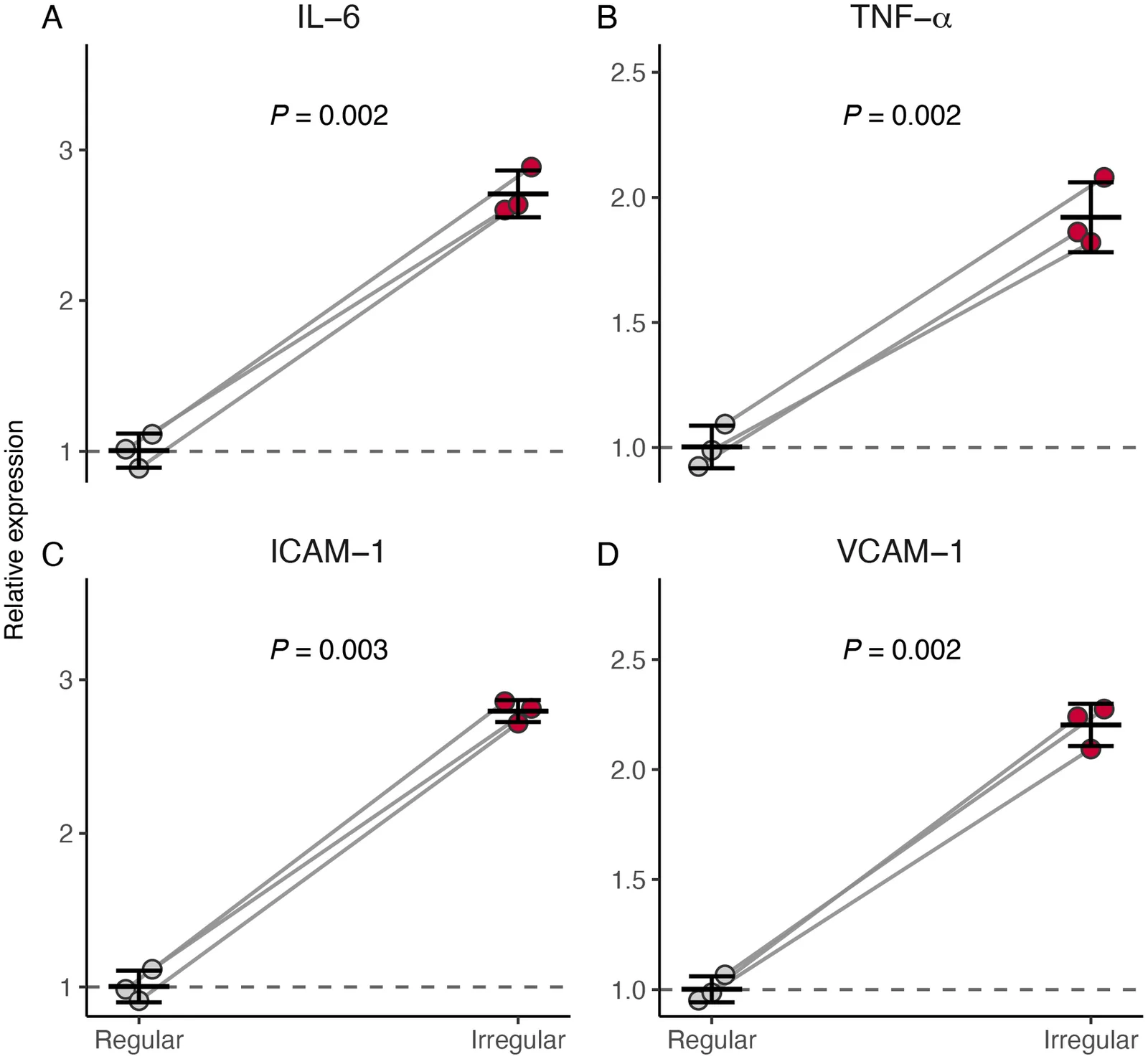
Inflammatory and adhesion-marker expression under regular and irregular dexamethasone schedules. Relative messenger RNA (mRNA) expression of **(A)** interleukin-6 (IL-6), **(B)** tumor necrosis factor-α (TNF-α), **(C)** intercellular adhesion molecule-1 (ICAM-1), and **(D)** vascular cell adhesion molecule-1 (VCAM-1) in human umbilical vein endothelial cells (HUVECs) subjected to the regular dexamethasone schedule or the four prespecified irregular schedule variants. Each point represents the run-level condition summary from one independent experimental run (*n* = 3). Within each run, the regular summary was the mean of four identically scheduled wells, whereas the irregular summary was the mean response across four prespecified schedule variants. Points from the same run are connected by gray lines; black horizontal bars and error bars indicate the mean ± standard deviation (SD) across runs. Expression values were normalized to one gene-specific common calibrator, defined as the mean *Δ*Ct of the regular condition across all three runs, thereby preserving run-to-run variation in the regular condition. Ct denotes the cycle threshold. The dashed line indicates relative expression = 1. *P* values were calculated using two-sided one-sample *t* tests of the run-level *ΔΔ*Ct values against 0. The experiment was limited to three independent runs with common-endpoint sampling; schedule-related and phase-related effects therefore could not be separated.

## 4 Discussion

In this nationally representative US cohort, greater night-to-night variability in sleep timing and duration was associated with higher hazards of all-cause mortality. Rubin-pooled estimates indicated 22%, 19%, and 17% higher all-cause mortality hazards per 1-hour increase in the across-night SD of sleep midpoint, sleep onset, and total sleep time, respectively. The across-night SDs of sleep midpoint and sleep onset were also associated with CVD mortality, whereas the total sleep time variability estimate was not statistically significant and remained uncertain. Lower SRI was associated with higher hazards of both outcomes. In three independent HUVEC runs, the mean response across the prespecified irregular schedule variants showed lower clock-gene expression and Griess assay absorbance and higher inflammatory and adhesion-marker expression than the regular schedule.

Two features extend the existing evidence. First, the population analysis identified sleep onset variability as an independent mortality correlate alongside sleep midpoint variability, total sleep time variability, and SRI, with effect sizes reported on both hourly and standardized scales. Second, the epidemiologic findings were complemented by independently repeated endothelial experiments showing coordinated changes in clock-gene expression, Griess assay absorbance, and inflammatory and adhesion markers. These components address different levels of evidence: the cohort quantifies population associations, whereas the cell model examines biological plausibility without establishing mediation of the population associations or causality in humans.

Our findings are consistent with prospective evidence linking sleep regularity to survival and cardiovascular health (17–20). The spline curves showed no evidence of nonlinearity, suggesting gradual changes across the observed range rather than a discrete threshold. The complementary category of sleep onset SD >90 min was informed by a previous prospective cardiovascular study rather than derived from the spline curve in the present analysis (19), and its association with all-cause mortality requires confirmation in independent cohorts.

Sleep onset variability, defined as the within-person SD of sleep onset across nights, was associated with all-cause mortality (HR, 1.19 per 1-hour increase; 95% CI, 1.11–1.29; *P* < 0.001). Because 1 h approximated the between-participant SD of this variability metric (0.93 h), the per-hour and standardized estimates were similar (standardized HR, 1.18). Prior prospective studies have examined sleep onset variability and composite sleep-regularity measures in relation to mortality (18, 20); the present findings extend this evidence to a nationally representative US cohort using a distinct accelerometer platform and sleep-scoring approach. Sleep onset timing may be behaviorally modifiable, but intervention studies are needed before this observational association can support clinical recommendations (9).

Several biological pathways may explain the observed associations. Night-to-night variability in sleep timing produces recurrent misalignment between the behavioral sleep–wake cycle and the endogenous circadian clock, disrupting the temporal coordination of cortisol secretion, sympathovagal balance, and vascular reactivity (16, 32, 33). Variable sleep timing also implies variable patterns of light exposure, meal timing, and physical activity, each of which entrains peripheral clocks in cardiovascular and metabolic tissues (19, 34). Chronic desynchrony of these entraining signals may promote endothelial dysfunction, systemic inflammation, and metabolic dysregulation—established precursors to atherosclerosis and premature death (35, 36). Standardized estimates changed little after adjustment for participant mean sleep duration, suggesting that sleep regularity may provide information beyond total sleep time. Nevertheless, established predictors such as older age, hypertension, and diabetes had larger associations in the complete models; the sleep measures should therefore be viewed as additional behavioral markers rather than dominant mortality predictors.

The HUVEC findings provide complementary evidence for the biological plausibility of these pathways. Compared with the regular schedule, the mean response across the prespecified irregular schedule variants showed lower BMAL1 and PER2 expression at the common sampling time, lower Griess assay absorbance, and higher expression of inflammatory and adhesion markers across three independent runs. Dexamethasone phase-resets peripheral cellular clocks (28, 29), but repeated dexamethasone exposure is not equivalent to spontaneous sleep irregularity in humans. The model isolates a schedule-related cellular perturbation while omitting the behavioral, neural, hormonal, metabolic, and environmental features of human sleep–wake disruption. Because all samples were collected at 168 h, the interval between the final stimulation and sampling also differed between the regular and irregular schedules; phase-related effects therefore cannot be separated from effects of schedule irregularity. The experiment lacked time-course sampling and clock-gene rescue, so the observed cellular differences cannot be assigned specifically to BMAL1 or PER2 suppression. Because the Griess assay was evaluated as blank-corrected absorbance without a nitrite standard curve or independent normalization to cell number, protein, or DNA, and CCK-8 absorbance was also lower, the Griess result should be interpreted as a semiquantitative nitrite-related signal rather than a specific measure of endothelial nitric oxide production. Future studies should include matched-phase sampling, genetic or pharmacologic rescue, and parallel measurement of circulating endothelial or inflammatory biomarkers.

Subgroup estimates were generally directionally consistent. After multiplicity correction, the associations of sleep midpoint and sleep onset variability with CVD mortality differed by marital status, whereas the remaining interaction tests were not statistically significant. These marital-status patterns may reflect psychosocial and socioeconomic differences associated with sleep regularity or other residual confounding (37, 38); they require replication because the subgroup analyses were exploratory and CVD event counts were limited. The smoking-status and joint-stratum results remain hypothesis-generating.

The present analysis extends previous NHANES studies of sleep regularity by examining prospective all-cause and CVD mortality. The survey-weighted mean SRI in our cohort was 61.96, and 65.8% of participants had SRI <70; this distribution was broadly consistent with other studies of US adults (37–39). By comparison, a UK Biobank study reported a median SRI of 81.0 (interquartile range, 73.8–86.3), with approximately one fifth of participants below 71.6 (30). Likewise, 42.6% of our cohort had an across-night sleep onset SD >90 min, compared with 25.6% in the Multi-Ethnic Study of Atherosclerosis (19). These differences may reflect population composition, recording windows, non-wear handling, and sleep-scoring algorithms and should not be interpreted as direct prevalence comparisons. Category-based HRs, especially those using the uncommon SRI ≥80 group as the reference, were imprecise and do not establish clinical risk thresholds.

Within the Life's Essential 8 framework, sleep-health counseling can address regularity together with adequate duration (6, 40). For adults with stable schedules, maintaining similar sleep-onset and wake times across workdays and days off is a reasonable behavioral goal (9). The category of sleep onset SD >90 min offers a simple descriptive benchmark for communication, but the present spline and categorical analyses do not validate it as a treatment target. For shift workers, recommendations should emphasize protected sleep opportunity and workplace scheduling strategies that limit abrupt or fatigue-promoting changes rather than applying a fixed clock-time target that may be infeasible (41). For middle-aged and older adults and those with chronic disease, sleep timing should be interpreted alongside symptoms, medications, sleep duration, and possible sleep disorders (40). These recommendations are implementation considerations rather than interventions tested in this study, and whether improving regularity reduces clinical events remains unknown.

Strengths of this study include objective sleep measurement with up to 7 consecutive days of continuous wrist accelerometry; circular statistics for clock-time SD; a noon-anchored analytic day; the NHANES survey design with population-level inference; and mortality ascertainment through the NDI.

Several limitations relate to sleep measurement. Partial capture of a sleep episode could distort estimated sleep onset, midpoint, or duration and introduce measurement error into the corresponding variability metrics, although the direction of bias is uncertain. Short recording windows may miss occasional timing deviations and incompletely characterize weekday–weekend differences when only one weekend day is available (42); limited observations can also reduce the stability of SD-based variability measures (27, 43). The 7-day analysis provided a complementary robustness check, but longer and repeated recordings would better characterize habitual regularity. The representative actograms provide illustrative examples of the sleep–wake patterns underlying different SRI values. The observed SRI distribution may also have been influenced by the recording window and scoring protocol (42). Accelerometer-based sleep–wake classification estimates behavioral sleep patterns from movement but does not measure sleep architecture or respiratory events (44).

The observational design precludes causal inference, and reverse causation and residual confounding by shift or night work, sleep disorders including sleep apnea, depression or other psychiatric conditions, chronic pain, occupational constraints, psychosocial circumstances, and other unmeasured factors remain possible. The approximate E-values were modest and should be interpreted as sensitivity measures rather than corrections for residual confounding (31). A negative-control analysis was not feasible because the linked dataset lacked a suitable exposure or outcome. The linked files ascertain mortality rather than incident nonfatal CVD, and the 229 CVD deaths limited precision for endpoint-specific and interaction analyses. Future cohorts should combine repeated long-term wearable monitoring with measurements of sleep apnea, mental health, occupational schedules, nonfatal cardiovascular outcomes, and circulating biomarkers. Mechanistic studies require matched-phase sampling and clock-gene rescue, and the clinical value of improving sleep regularity ultimately requires randomized intervention studies.

## 5 Conclusion

Greater night-to-night sleep variability and lower sleep–wake regularity were associated with higher hazards of all-cause and CVD mortality in this nationally representative US cohort. Sleep onset variability and SRI may be useful markers for sleep-health research, but the observational findings do not establish causality or clinical thresholds. Whether interventions that improve sleep-timing consistency reduce cardiovascular events or mortality requires randomized testing.

## Data Availability

The datasets presented in this study can be found in online repositories. The names of the repository/repositories and accession number(s) can be found in the article/Supplementary Material.
